# Expression of PD-1 on CD4^+^ Tumor-Infiltrating Lymphocytes in Tumor Microenvironment Associated with Pathological Characteristics of Breast Cancer

**DOI:** 10.1155/2018/5690258

**Published:** 2018-07-04

**Authors:** Yan-Jie Zhao, Jian Zhang, Feng Shi, Zhi-Ping Hu, Jiang-Ping Wu, Guang-Jiang Wu, Rui-Bin Wang, Quan Zhou, Hong Chang, Ying-Nan Li, Qing-Kun Song

**Affiliations:** ^1^Department of Medical Oncology, Beijing Shijitan Hospital, Capital Medical University, Beijing 100038, China; ^2^Department of Emergency, Beijing Chaoyang Hospital, Capital Medical University, Beijing 100020, China; ^3^Department of Pathology, Beijing Shijitan Hospital, Capital Medical University, Beijing 100038, China; ^4^Department of Hepatobiliary Surgery, Peking University People's Hospital, Beijing 100021, China; ^5^Department of Cancer Research, Beijing Shijitan Hospital, Capital Medical University, Beijing 100038, China; ^6^Department of Infection Control, Beijing Shijitan Hospital, Capital Medical University, Beijing 100038, China; ^7^Department of Emergency, Beijing Shijitan Hospital, Capital Medical University, Beijing 100038, China; ^8^Department of Geriatric Gastroenterology, Chinese PLA General Hospital, Beijing 100853, China; ^9^Department of Science and Technology, Beijing Shijitan Hospital, Capital Medical University, Beijing 100038, China; ^10^Beijing Key Laboratory of Cancer Therapeutic Vaccine, Beijing, China

## Abstract

**Objective:**

This study aimed to investigate the correlation of CD4^+^/PD-1^+^ or CD4^+^/PD-1^−^ tumor-infiltrating lymphocytes with pathological characteristics in breast cancer patients.

**Methods:**

A cross-sectional study consecutively recruited 133 patients with invasive ductal breast cancer. The expression of CD4, programmed cell death protein 1 (PD-1), CK7, CK20, E-cadherin, or Ki-67 was detected by immunohistochemistry. The associations between CD4^+^/PD-1^+^ or CD4^+^/PD-1^−^ tumor-infiltrating lymphocytes and pathological characteristics were evaluated.

**Results:**

Elderly patients intended to have a lower level of CD4^+^/PD-1^−^ tumor-infiltrating lymphocytes (*p* < 0.05). Patients with positive E-cadherin expression had higher median cell counts of CD4^+^/PD-1^−^ tumor-infiltrating lymphocytes than patients with negative E-cadherin expression (30/HPF versus 10/HPF, *p* < 0.05). Counts of CD4^+^/PD-1^+^ tumor-infiltrating lymphocytes had a significant correlation with Ki-67 index that the correlation coefficient was 0.29 (*p* = 0.001). Positive CK20 expression was related to a higher level of CD4^+^/PD-1^−^ tumor-infiltrating lymphocytes than negative CK20 expression (73/HPF versus 30/HPF, *p* < 0.05).

**Conclusion:**

CD4^+^/PD-1^+^ or CD4^+^/PD-1^−^ tumor-infiltrating lymphocytes showed diverse association with pathological features of breast cancer. CD4^+^/PD-1^+^ tumor-infiltrating lymphocytes had a significant relationship with Ki-67 expression whereas CD4^+^/PD-1^−^ tumor-infiltrating lymphocytes had a significant relationship with E-cadherin expression. Further studies are warranted to explore the immunomodulatory effects of phenotypes of CD4^+^ T cell subsets in breast cancer.

## 1. Introduction

Breast cancer (BC) is the most common female cancer worldwide with 1.67 million new cases (25% of all incident cancer cases) in 2012 [1], and the mortality rate was higher in underdeveloped countries than in developed countries [1]. Recently, tumor-infiltrating lymphocytes (TILs) were reported to be involved in BC development, prognosis, and immunotherapy efficacy.

TILs are mainly composed of different subtypes of T cells, which play an important role in antitumor immunity. A high proportion of TILs predicted a favorable prognosis of BC patients [2]. CD4^+^ T lymphocytes are helper T cells, and high counts of intratumoral CD4^+^ lymphocytes conferred a better BC survival [3]. Programmed cell death protein 1 (PD-1), as an immune checkpoint expressed on T cells, binds to programmed cell death ligand 1 (PD-L1) on the surface of cancer cells and suppresses antitumor functions of T lymphocytes. So the expression of PD-1 indicated exhausted function of lymphocytes and a high level of PD-1^+^ TILs correlated with a worse survival of BC [4]. According to PD-1 expression, CD4^+^ lymphocytes can be further classified into two subgroups, subsets of CD4^+^/PD-1^+^ and CD4^+^/PD-1^−^ T lymphocytes. The immune function of CD4^+^ T cells was affected by PD-1 expression. The immune function of CD4^+^/PD-1^−^ TILs was less exhausted than that of CD4^+^/PD-1^+^ TILs [5]. Among the HIV-infected patients, CD4^+^/PD-1^+^ T lymphocytes played an important role in the HIV persistence and immunomodulatory microenvironment [6, 7]. However, very few studies were focused on exhausted CD4 T cells in BC patients. Cytokeratins 7 (CK7) and 20 (CK20) were related to the pathological features of BC [8]. E-cadherin (E-Cad) and Ki-67 showed a significant relationship with BC prognosis [9]. These biomarkers were tested routinely in BC pathological diagnosis. We aimed to investigate the correlation of exhausted status of CD4+ helper T cells with pathological characteristics among BC patients.

## 2. Methods

### 2.1. Ethical Approval

All procedures performed in this study involving human participants were approved by the ethical committee of Beijing Shijitan Hospital, Capital Medical University, in accordance with the ethical standards of the 1964 Helsinki declaration and its later amendments.

### 2.2. Informed Consent

As a retrospective study, the formal consent was waivered.

#### 2.2.1. Patients

This cross-sectional study included 133 patients with invasive ductal BC. Patients were diagnosed with operable BC and received surgical treatment at the Department of Breast Surgery, Beijing Shijitan Hospital, Capital Medical University, consecutively from January 1, 2012, to December 31, 2013. All the cases were pathologically confirmed with primary invasive BC.

#### 2.2.2. Tissue Collection

Archival formalin-fixed, paraffin-embedded (FFPE) BC samples were collected from all patients. The surgical specimen was fixed by 4% neutral formaldehyde, embedded by paraffin, and stained by hematoxylin and eosin. Histopathologic feature was determined using serial 4 *μ*m thickness sections derived from each specimen. Nottingham modification of the Bloom-Richardson system was used to classify histological grading of BC.

*(1) Immunohistochemistry (IHC)*. Expression profiling of PD-1, CK7, CK20, Ki-67, E-Cad, and CD4 was assessed by IHC on 4 *μ*m thick FFPE sections. Monoclonal antibodies against PD-1 (mouse anti-human, #UMAB199), CD4 (rabbit anti-human, #EP204), CK7 (rabbit anti-human, #EP16), CK20 (rabbit anti-human, #EP23), Ki-67 (mouse anti-human, #MIB1), and E-cadherin (E-Cad) (mouse anti-human, #NCH-38) were purchased from Beijing Zhong Shan Golden Bridge Biotechnology Co., Ltd. Sections were baked at 60°C in a dehydration oven for 60 min, dewaxed for 20 min, and washed in graded alcohol of 100%, 100%, 95%, and 75% for 2 min, respectively. Sections were washed with PBS for 2 min by 5 times. Antigen retrieval was carried out using the EnVision™ FLEX Target Retrieval Solutions for 2 min 30 sec, cooled to room temperature for 20 min, washed with PBS for 2 min by 5 times, incubated with 3% H_2_O_2_ for 15 min at room temperature, washed with PBS for 2 min by 5 times, sealed with 5% serum at 37°C for 15 min, discarded, and added a moderate primary antibody at 4°C for a night. With PBS wash for 2 min by 5 times, DAB was added for 5–10 min and AP-red was added for 10–15 min. PD-1, CK7, CK20, Ki-67, and E-Cad detection were visualized with DAB whereas CD4 was visualized with AP-red. Slides were counterstained with hematoxylin.

TILs were counted in randomly selected five different high-power fields (HPF) to obtain an average number in IHC sections by two pathologists. PD-1 was expressed in the cytoplasm of lymphocytes with the color of brown, CK7 and CK20 were expressed in the cytoplasm of BC cells with the color of brown, Ki-67 was expressed in nucleus of BC cells with color of brown, E-Cad was expressed on cytomembrane of BC cells with the color of brown, and CD4 was expressed on the cytomembrane of lymphocytes with the color of red. Double staining of CD4/PD-1 showed red cytomembrane and brown cytoplasm of lymphocytes. We counted the PD-1- or CD4-positive cells in 100 lymphocytes and calculated the expression rate. We counted PD-1-positive cells among 100 CD4^+^ lymphocytes and calculated the proportion. Ki-67 index was estimated among 100 BC cells. Ki-67 index > 1% was defined as positive expression.

### 2.3. Statistical Analysis

All analyses were conducted with SPSS software (version 17.0). Median and interquartile range (IQR) were used to describe TIL counts. Age was transformed in categorical scale by median of 55. The comparisons of TIL phenotypes were processed by Wilcoxon tests between age, nerve invasion, vascular invasion, and axillary lymph node involvement groups. The association of TIL phenotypes with histological grade was estimated by Spearman correlation tests. Wilcoxon tests were used to estimate the difference of TIL phenotypes between positive and negative expression of CK7, CK20, and E-Cad. Spearman correlation test was used to measure the relationship between Ki-67 index and cell counts of TIL phenotypes. All analyses were two sided, and a significant level was 0.05.

## 3. Results

### 3.1. General Characteristics

The average age of included patients was 57.8 years old (Table 1). 11.3% patients were diagnosed at histological grade I, 32.3% patients had vascular invasion, 17.3% patients had nerve invasion, and 54.2% patients had axillary lymph node metastasis (Table 1). Average Ki-67 index was 30% (Table 1). 94.9%, 8.2%, and 86.8% patients had positive expression of E-Cad, CK20, and CK7, respectively (Table 1). The median counts of PD-1^+^ TILs was 18/HPF.

### 3.2. CD4/PD-1 TILs and Clinical Characteristics

**Figure 1:**
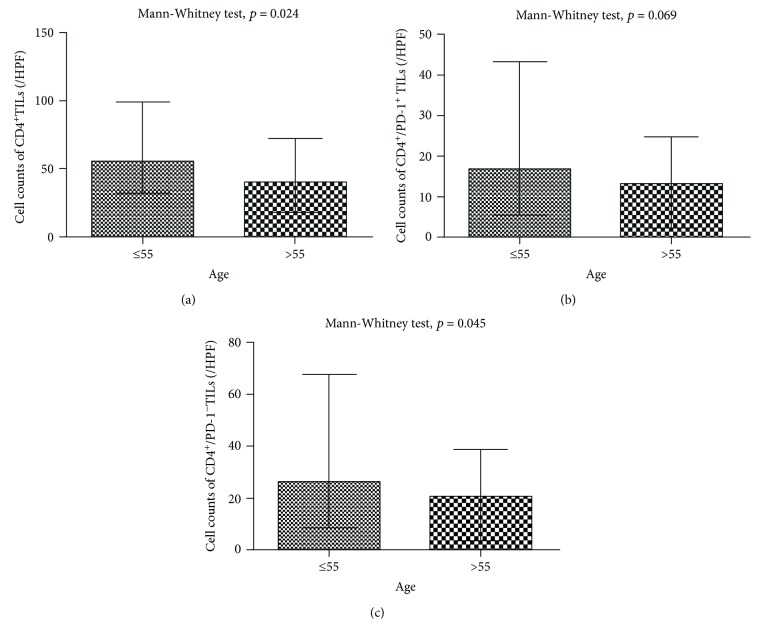
Relationship between age and CD4/PD-1 phenotypes of TILs. (a) Relationship between age and CD4^+^ TILs. (b) Relationship between age and CD4^+^/PD-1^+^ TILs. (c) Relationship between age and CD4^+^/PD-1^−^ TILs.

Age was significantly associated with the number of CD4^+^ TILs: the median of CD4^+^ TILs was 56/HPF and 40/HPF in the group of age equal to or younger than 55 and older than 55, respectively (*p* < 0.05, Figure 1(a), Table 2). The median count of CD4^+^/PD1^−^ TILs was 32/HPF among cases equal to or younger than 55 and 25/HPF among cases older than 55, respectively (*p* < 0.05, Figure 1(c), Table 2). Histological grade, vascular invasion, nerve invasion, or axillary lymph node involvement had no association with phenotypes of CD4^+^ TILs (Table 2).

### 3.3. CD4/PD-1 TILs and Other Molecules

**Figure 2:**
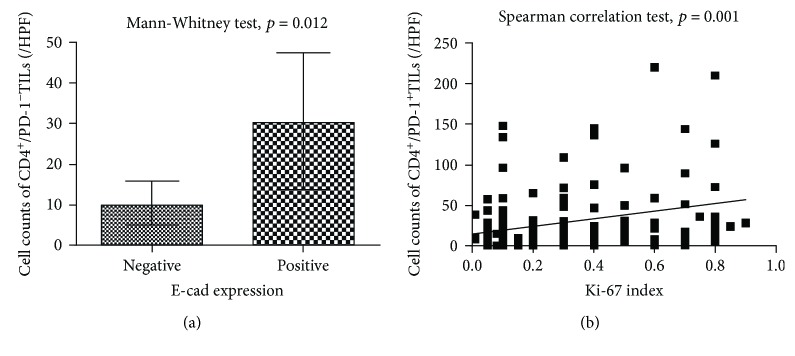
Association between CD4/PD-1 TILs, E-Cad expression, and Ki-67 index. (a) Association between E-Cad expression and CD4^+^/PD-1^−^ TILs. (b) Correlation between Ki-67 index and CD4^+^/PD-1^+^ TILs.

**Figure 3:**
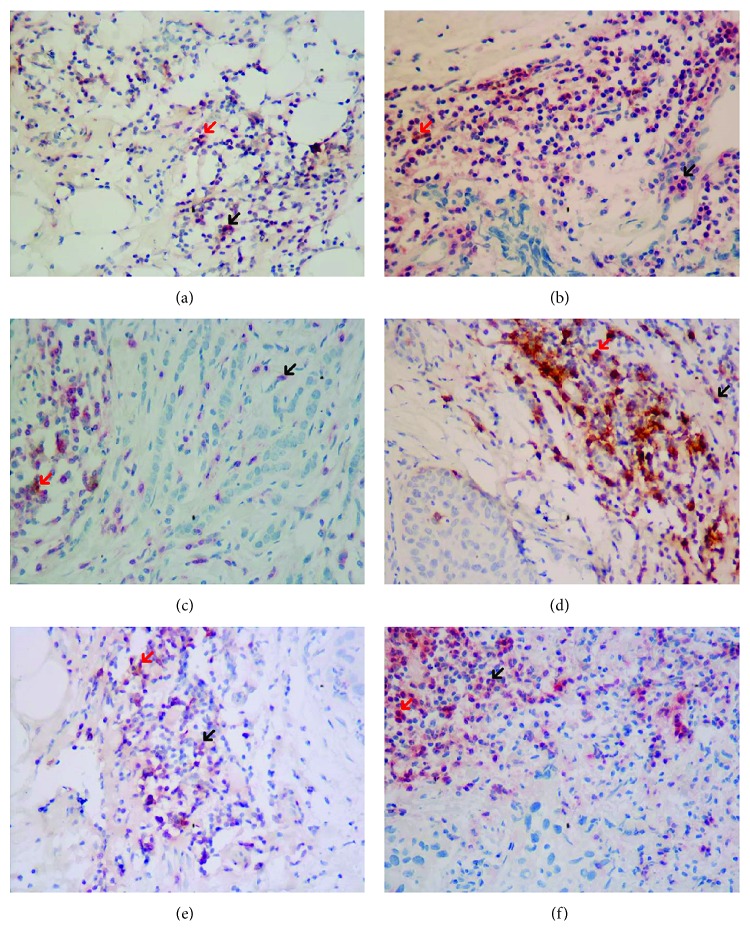
Expression of E-Cad, Ki-67, CK20, and counts of phenotypes of CD4/PD-1 TILs. (a) Patients with negative E-Cad expression (IHC, ×400). (b) Patients with positive E-Cad expression (IHC, ×400). (c) Patients with negative Ki-67 expression (IHC, ×400). (d) Patients with positive Ki-67 expression (IHC, ×400). (e) Patients with negative CK20 expression (IHC, ×400). (f) Patients with positive CK20 expression (IHC, ×400). →CD4^+^/PD-1^+^ TILs; →CD4^+^/PD-1^−^ TILs. CD4^+^/PD1^+^ TILs showed red cytomembrane and brown cytoplasm, and CD4^+^/PD1^−^ TILs showed red cytomembrane. Patients with negative E-Cad had less CD4^+^/PD-1^−^ TILs than those with positive E-Cad; patients with negative Ki-67 had less CD4^+^/PD-1^+^ TILs than those with positive Ki-67; patients with negative CK20 expression had less CD4^+^/PD-1^−^ TILs than those with positive CK20 expression.

E-Cad expression had a significant association with CD4^+^ TIL counts: the median count of CD4^+^ TILs was 23/HPF and 48/HPF among patients with negative and positive E-Cad expression, respectively (*p* < 0.05, Table 3). Cell count of CD4^+^/PD1^−^ TILs, not CD4^+^/PD1^+^ TILs, was significantly related to E-Cad expression: the median count of CD4^+^/PD-1^−^ TILs was 10/HPF and 30/HPF among patients with negative and positive E-Cad expression, respectively (*p* < 0.05, Figure 2(a), Table 3). The correlation coefficient was 0.20 (*p* < 0.05) between Ki-67 index and the count of CD4^+^ TILs (Table 3). Ki-67 index was significantly related to the cell counts of CD4^+^/PD1^+^ TILs (Table 3), with a coefficient of 0.29 (*p* < 0.05, Figure 2(b), Table 3). CD4^+^/PD1^+^ TILs showed red cytomembrane and brown cytoplasm, and CD4^+^/PD1^−^ TILs showed red cytomembrane (Figure 3). Patients with negative E-Cad expression (Figure 3(a)) had less CD4^+^/PD-1^−^ TILs in tumor microenvironment than those with positive E-Cad expression (Figure 3(b)). Patients with positive Ki-67 expression (Figure 3(c)) had more CD4^+^/PD1^+^ TILs than those with negative Ki-67 expression (Figure 3(d)). CK20 expression percentile was significantly associated with CD4^+^/PD1^−^ TIL cell count (Table 3). The median of CD4^+^/PD-1^−^ TILs was 30/HPF in the group of negative CK20 expression, significantly lower than 73/HPF in that of positive CK20 expression (Table 3). In the pathological slides, cell counts of CD4^+^/PD-1^−^ TILs in the group of negative CK20 expression (Figure 3(e)) were lower than those of positive CK20 expression (Figure 3(f)). Expression of CK7 was not associated with any phenotypes of CD4^+^ TILs (Table 3).

## 4. Discussion

Despite the development in therapies for BC, there is a gap in clinical efficacy between Chinese and US patients. The immune system is critically involved in tumor surveillance and development. Immunotherapy is a highly attractive alternative approach to treat patients with advanced BC [10].

An increased number of TILs was related to a better clinical outcome of cancer patients [11]. The dynamic change and dysfunction of TIL subsets in the immunosuppressive microenvironment contribute to the pathogenesis of cancer. A better understanding of TILs may facilitate the development of effective strategies for immunotherapy. An effective antitumor immune response requires both CD4^+^ and CD8^+^ T lymphocytes [12, 13]. CD8^+^ T lymphocytes always play a critical role in antitumor immunity. The function of CD4^+^ T lymphocytes in cancer has recently been extensively studied in both animal models and patients. CD4^+^ T lymphocytes typically recognize peptides with the size of 12–16 AA, presented by MHC class II molecules [14]. These lymphocytes are important for the initiation and maintenance of adaptive immune responses. The subsets of CD4^+^ T lymphocytes had diverse antitumor immunity. The change of CD4^+^ T lymphocytes from Th1 to Treg and Th17 cells affected the immune functions [15].

Thrombocytopenia patients and chronic lymphocytic leukemia patients had a higher level (in both percentage and absolute number) of CD4^+^/PD1^+^ lymphocytes than of healthy controls [16–19]. The particular subsets of CD4^+^ lymphocytes had reduced proliferation ability and produced less amounts of IL-1, TNF-*α*, IL-10, and IFN-*γ* [17]. CD4^+^/PD-1^+^ T lymphocytes were found to be in close contact with PD-L1^+^ chronic lymphocytic leukemia cells [19] and the activated PD-1/PD-L1 axis led to decreased production of IL-4 from CD4^+^ T lymphocytes [19]. High level of CD4^+^/PD1^+^ lymphocytes, reduced cytokine secretion, dysfunctional proliferation, and increased apoptosis were also observed among sarcoidosis patents [20, 21], but blocking PD-1 pathway in CD4^+^ T cells could reverse the proliferative capacity [21]. The expression of PD-1 on CD4^+^ lymphocytes indicated exhausted function [20, 21]. CD4^+^/PD1^+^ TILs were dysfunctional in the presence of PD-L1 among patients with head and neck squamous cell carcinomas [22] and glioblastoma multiforme (GBM) [5]. Though the immune function of CD4^+^/PD-1^+^ T cells was exhausted in tumor microenvironment, CD4^+^/PD-1^−^ effector TILs were more metabolically active and proliferative and enriched in immune costimulation gene sets than CD4^+^/PD-1^+^ effector TILs from GBM [5].

PD-1 expression on CD4^+^ T cells contributed to the dysfunction of T cells. Compared with healthy controls, the expression of PD-1 on peripheral CD4+ T cells was decreased among psoriatic and rheumatoid arthritis (RA) patients [23, 24]. High expression of CD4^+^/PD-1^+^ T cells was related to a reduced activity score of RA [24]. This phenomenon reflected a negative regulation of immune response by CD4^+^/PD-1^+^ lymphocytes in the pathogenesis of psoriasis and RA.

Elderly BC patients had more favorable prognostic factors than younger ones, such as higher grading score and hormone receptor expression; however, their survival was not as good as expected [25]. Hormone receptor positivity was increased from 60% in patients aged 30–35 to 85% in patients aged 80–85 [26]. About 60–70% of all BC patients were diagnosed at early stages; however, their prognosis varies greatly [27]. The variation in immune system of elderly patients accounted for the difference to some extent. In our study, the median of CD4^+^ TILs was 40/HPF in elderly BC patients, lower than in younger patients (56/HPF). Age was significantly associated with the number of CD4^+^/PD1^−^ TILs. The median of CD4^+^/PD1^−^ TILs was 32/HPF among cases equal to or younger than 55 and 25/HPF among those older than 55, implying the degradation of immune function among elderly patients.

The E-Cad, an important member of cell adhesion molecule family, is encoded by the cadherin gene located on chromosome 16q22.1. The E-Cad mediates molecular recognition and binding, tumor infiltration, and metastasis [28]. Low expression of E-Cad was related to a poorer recurrence-free survival of invasive ductal BC [29]. Our study revealed that E-Cad expression was significantly associated with the number of CD4^+^/PD-1^−^ TILs. BC cells expressing more E-Cad recruited more functional CD4^+^ TILs in tumor microenvironment.

Ki-67 is a cell cycle-dependent nuclear antigen and illustrates the proliferative activity of cells [30, 31]. Expression rate of Ki-67 was regarded as a proliferation index in cancer cells [32]. We identified a correlation coefficient of 0.29 between Ki-67 index and the count of CD4^+^/PD-1^+^ T cells. A high Ki-67 index implied an exhausted status of tumor microenvironment. Thus, Ki-67 acted as a negative prognostic and predictive marker for BC patients [33–36].

A small sample size was one limitation of our study. In addition, we did not analyze intratumoral and stromal TILs separately. Not using flow cytometry to detect TILs phenotypes was another flaw.

## 5. Conclusion

Among BC patients, CD4^+^/PD-1^+^ and CD4^+^/PD-1^−^ TILs had diverse pathological features in tumor microenvironment. The number of CD4^+^/PD-1^+^ TILs was significantly related to Ki-67 expression, while the level of CD4^+^/PD-1^−^ TILs significantly correlated with E-Cad expression. PD-1 expression on CD4^+^ TILs indicated dysfunctional immune microenvironment. Further studies are warranted to explore the immunotherapy for BC patients.

## Figures and Tables

**Table 1:** The characteristics of patients.

Items	
Age, mean ± SD	57.8 ± 13.6
Histological grade, *n* (%)	
I	14 (11.3)
II	82 (66.1)
III	28 (22.6)
Vascular invasion, *n* (%)	
No	86 (67.7)
Yes	41 (32.3)
Nerve invasion, *n* (%)	
No	101 (82.1)
Yes	22 (17.3)
Axillary lymph node metastasis, *n* (%)	
No	22 (45.8)
Yes	26 (54.2)
E-cadherin expression, *n* (%)	
No	6 (5.1)
Yes	112 (94.9)
Ki-67 index, mean ± SD	30% ± 25%
CK20 expression, *n* (%)
No	56 (91.8)
Yes	5 (8.2)
CK7 expression, *n* (%)	
No	9 (13.2)
Yes	59 (86.8)

**Table 2:** The relationship between CD4/PD-1 TILs and clinical characteristics.

	CD4^+^TILs	*p*	CD4^+^/PD1^+^TILs	*p*	CD4^+^/PD1^−^TILs	*p*
Age, median (IQR)^∗^		0.024		0.069		0.045
≤55	56 (67)		17 (38)		32 (43)	
>55	40 (54)		13 (22)		25 (29)	
Histological grade, median (IQR)^∗∗^		0.083		0.136		0.092
I	40 (48)		13 (29)		17 (26)	
II	45 (65)		13 (32)		32 (33)	
III	55 (82)		22 (40)		32 (58)	
Vascular invasion, median (IQR)		0.316		0.914		0.275
No	42 (63)		15 (32)		26 (35)	
Yes	54 (47)		16 (25)		32 (22)	
Nerve invasion, median (IQR)^∗^		0.601		0.826		0.365
No	44 (48)		16 (28)		27 (28)	
Yes	54 (88)		14 (44)		32 (53)	
Axillary lymph node metastasis, median (IQR)^∗^		0.469		0.725		0.169
No	67 (71)		20 (26)		50 (47)	
Yes	57 (48)		14 (45)		30 (33)	

^∗^Wilcoxon test. ^∗∗^Spearman correlation test.

**Table 3:** Correlation between CD4/PD-1 TILs and other molecules in breast cancer.

	CD4^+^ TILs	*p*	CD4^+^/PD1^+^ TILs	*p*	CD4^+^/PD1^−^ TILs	*p*
CK7, median (IQR)^∗^		0.913		0.724		0.684
No	72 (94)		14 (81)		26 (31)	
Yes	56 (76)		22 (44)		32 (52)	
CK20, median (IQR)^∗^		0.152		0.415		**0.025**
No	55 (84)		22 (43)		30 (45)	
Yes	100 (53)		31 (55)		73 (46)	
E-Cad, median (IQR)^∗^		**0.013**		0.157		**0.012**
No	23 (28)		5 (25)		10 (11)	
Yes	48 (63)		16 (32)		30 (34)	
Ki-67, correlation coefficient^∗∗^	0.20	**0.023**	0.29	**0.001**	0.09	0.296

^∗^Wilcoxon test. ^∗∗^Spearman correlation test.

## Data Availability

The data used to support the findings of this study are available from the corresponding author upon request.
